# Psychometric properties of the German version of the pregnancy-related anxiety questionnaire-revised 2 (PRAQ-R2) in the third trimester of pregnancy

**DOI:** 10.1186/s12884-019-2368-6

**Published:** 2019-07-11

**Authors:** S. Mudra, A. Göbel, D. Barthel, K. Hecher, M. Schulte-Markwort, J. Goletzke, P. Arck, A. Diemert

**Affiliations:** 1 Department of Child and Adolescent Psychiatry, Psychotherapy and Psychosomatics, University Medical Center Hamburg-Eppendorf, Martinistr. 52, 20246 Hamburg, Germany; 2 Department of Obstetrics and Fetal Medicine, University Medical Center Hamburg-Eppendorf, Martinistr. 52, 20246 Hamburg, Germany

**Keywords:** Pregnancy-related anxiety, PRAQ-R2, Psychometric properties, Prenatal, Anxiety

## Abstract

**Background:**

Pregnancy-related anxiety (PrA) has been identified as a construct distinct from general stress and anxiety with a negative impact on birth and child outcomes. Validated instruments with good psychometric properties to assess pregnancy-related anxiety in German-speaking expectant mothers are still lacking. The Pregnancy-Related Anxiety Questionnaire revised for its use independent of parity (PRAQ-R2) assesses fear of giving birth (FoGB), worries of bearing a physically or mentally handicapped child (WaHC) and concerns about own appearance (CoA). The aim of this study was to investigate the psychometric properties of the PRAQ-R2 in a German sample of pregnant women in their third pregnancy trimester.

**Methods:**

The PRAQ-R2 and several questionnaires measuring different forms of anxiety as well as depressive symptoms and perceived general self-efficacy were administered cross-sectionally in a sample of nulliparous and parous women (*N* = 360) in the third trimester of pregnancy.

**Results:**

Reliability was satisfactory to excellent for the PRAQ-R2 total scale (Cronbach’s *α* = .85) and the subscales (*α* = .77 to .90). Confirmatory and exploratory factor analysis confirmed the three-factorial structure of the instrument. The three factors together explained 68% of variance. Construct validity was confirmed by positive low- to moderate-sized correlations of the PRAQ-R2 total score and the subscales with measurements of anxiety and depression and by negative low correlations with general self-efficacy.

**Conclusions:**

The German version of the PRAQ-R2 is a valid and feasible measurement for pregnancy-related anxiety for research and clinical practice.

## Background

During the last decade, the research focus on maternal mental health has expanded from the postpartum to the prenatal period. Several studies provided evidence of a negative impact of prenatal maternal anxiety on pregnancy and birth outcome or infant developmental problems [1, 2]. Pregnancy-related anxiety (PrA), which centers around infant’s health, childbirth, and maternal bodily changes and appearance, has been identified as a construct distinct from general anxiety and anxiety disorders [3, 4]. Published evidence indicates that PrA was a strong predictor for birth and pregnancy-related outcomes (e.g. birth procedure, pregnancy complications), postpartum maternal mood and long-term child-related consequences (e.g. infant cerebral, cognitive and emotional development, temperament or behavioral outcomes) [3–12].

These results not only underline the clinical relevance of PrA but also emphasize the importance of a differentiated approach to assessing different forms of prenatal anxiety. Only a few instruments measure the broad construct of PrA sufficiently across pregnancy [13, 14]. Most instruments available in German assess specific aspects of PrA only, such as the scales of Lukesch [15] and Ringler [16] focusing on fear of childbirth or the Baby Schema Questionnaire [17] focusing on concerns about the child’s health. The German Cambridge Worry Scale (CWS; [18]) assesses birth- or child-specific worries besides other areas of potential prenatal concern. Nevertheless, widely used instruments focusing on the broader concept of PrA are not yet validated in German, which makes a transcultural comparison of PrA-related findings more difficult.

One of the globally most frequently used instruments, the Pregnancy-Related Anxiety Questionnaire (PRAQ [19];) has been revised by Huizink and colleagues [20] into a feasible abbreviated 10-item version (PRAQ-R) with the three subscales “fear of giving birth” (FOGB), “worries of bearing a physically or mentally handicapped child” (WaHC), and “concerns about own appearance” (CoA). To enable the use of the instrument regardless of parity the questionnaire has been recently adapted (PRAQ-R2; [21]) by rephrasing one item to “I am anxious about the delivery”. Psychometric properties of the PRAQ-R2 were assessed in nulli- and parous women from Finland in their 24th and 34th weeks of pregnancy [21]. Internal consistencies were high for the total score (nulliparous/parous 24th week α = .84/.82, 34th week α = .84/.85) and satisfactory to high for the subscales FoGB (nulliparous/parous 24th week α = .79/.71, 34th week α = .75/.75), WaHC (nulliparous/parous 24th week α = .77/.80, 34th week α = .80/.83) and CoA (nulliparous/parous 24th week α = .80/.80, 34th week α = .82/.81). Confirmatory factor analysis supported the three-factor solution for the PRAQ-R2 independent of parity and gestational age [21]. In current studies on French [22] and Turkish [23] versions of the PRAQ-R2, convincing psychometric properties were reported and the three-factor structure confirmed. Associations of PrA with different forms of anxiety and depression have been reported in prior literature. Moderately sized correlations between the PRAQ-R total score and the scales of the State-Trait Anxiety Inventory for state anxiety (STAI-S; *r* = .46 to .63) and trait anxiety (STAI-T; *r* = .46 to .60) were found [24–26]. On subscale level, correlations with STAI-S and STAI-T were comparable in size for FoGB (*r* = .26 to .39), WaHC (*r* = .27 to .39) and CoA (*r* = .24 to .33). Moderate associations of the PRAQ-R total score with the Edinburgh Postnatal Depression Scale (*r* = .36; [26]) and the Beck Depression Inventory have been reported (*r* = .37 to .51; [27]). In regression analyses, general levels of anxiety and depression explained only a small amount of variance in PrA, supporting the assumption of PrA as a distinct construct [20].

Blackmore et al. [3] found in the second and third trimester of pregnancy significant positive correlations of symptoms of generalized anxiety disorders (GAD) with FoGB (*r* = .12 to .24, respectively) and WaHC (*r* = .22 to .23, respectively). Martini et al. [28] investigated specific birth- and child-related fears in the perinatal period. They found that women with social phobia (SP) reported higher postpartum child-related anxiety than a control group.

General self-efficacy has been identified as a potential protective factor for PrA. Perceived self-efficacy is generally defined by Bandura [29] as a cognitive process, in which a person evaluates own capabilities to cope with different situations and to act in a way to master challenging situations. Empirical studies support the assumption of negative associations between perceived self-efficacy and anxiety [30]. Focusing on pregnancy and childbirth as potentially challenging situations, previous studies showed that women with higher fear of giving birth perceived their general and birth-related self-efficacy as lower [31]. Despite the fact that the PRAQ-R2 has proved to be a reliable instrument [21], a psychometric investigation of a German translation of the PRAQ-R2 is still lacking. Thus, the aim of this study was to investigate the reliability as well as factorial and construct validity (convergent and discriminant) for the PRAQ-R2 in a population-based sample of nulliparous and parous women from northern Germany in their third trimester of pregnancy.

Regarding convergent validity, we expected positive associations of the PRAQ-R2 with pregnancy-related worries as well as state and trait anxiety, depression and symptoms of GAD and SP according to previous research [3, 20, 28]. Regarding discriminant validity, we expected negative correlations of the PRAQ-R2 total score with perceived general self-efficacy in line with previous studies [30, 31].

## Methods

### Study design and sample

The data derive from two related ongoing population-based longitudinal pregnancy cohorts (PRINCE – “Prenatal Identification of Children’s Health” and PAULINE – “Prenatal Anxiety and Infant Early Emotional Development”) based at the University Medical Center Hamburg-Eppendorf.

Pregnant women were recruited upon initial presentation at the university after being sent by their resident gynecologists or midwifes between 2014 and 2018. Women pregnant with a singleton child and 18 years or older were included in this study. Women with high-risk pregnancies regarding maternal chronic infections, severe complications in mother or child, substance abuse, as well as women with a pregnancy after assisted reproductive technologies (ART) were excluded. Also, women lacking sufficient German language skills were excluded. All participants signed informed consent forms. There was no incentive to participate and study involvement was voluntary. The study protocols were approved by the ethics committee of the Hamburg Chamber of Physicians (PV3694, PV5574).

For PRAQ-R2 and further relevant psychometric questionnaires, full datasets were available for 253 pregnant women. To ensure statistical power for this analysis, PRAQ-R2 data of additional 107 women were pooled from the PRINCE-study. Overall, psychometric data of 360 women in the third trimester of pregnancy were analyzed.

### Variables and instruments

#### PRAQ-R2

The ten PRAQ-R2 items are scored with five response options (1 = “absolutely not relevant” to 5 = “very relevant”). A total score (range from 10 to 50) and one score for each of the subscales FoGB (3 items, range from 3 to 15), WaHC (4 items, range from 4 to 20) and CoA (3 items, range from 3 to 15) can be calculated.

The PRAQ-R2 [21] was translated into German for the purpose of this study following the recommendations by Bracken and Barona [32]. Permission to use the PRAQ-R2 was given by the author, Professor Anja Huizink. The English version was independently translated into German by two members of the study team. Based on these two translations, a final German version was developed. Minimal differences in wording were discussed with the PI of the study until consent was reached. This German version was back-translated from German by two blinded independent expert linguists without knowledge about the study content and the original questionnaire. A comparison of the back-translated versions with the original questionnaire revealed no semantic change in the items due to the translation process. Finally, the German version was handed out to two pregnant and two non-pregnant women, who were not familiar with the content of the study and reported no difficulties in understanding the items.

#### Instruments for the assessment of convergent validity

- Pregnancy-specific worries: The CWS [33] assesses prenatal anxiety with 17 items rated on a 5-point scale and with mean scale scores ranging from 0 to 5. The subscales “socio-medical” (centering around birth and handling of the baby) and “health of the baby” show similarities to FoGB and WaHC. The subscales “socio-economic and relations” and “health of mother/other” focus on aspects of the social environment and living circumstances as parents. We excluded one item regarding employment problems, which was irrelevant for our participants at this time of pregnancy due to legally binding maternity leave in Germany, starting 6 weeks before the estimated time of delivery. In our sample, reliability was good for the total score (Cronbach’s α = .82) and satisfactory for the subscales (α = .60 to .76).
- General State and Trait Anxiety: State and trait anxiety were assessed with the STAI [34], which consists of two 20-item subscales (STAI-S/−T). Items are rated on a 4-point scale so that scale scores range from 20 to 80 (STAI-S Cronbach’s α = .94; STAI-T Cronbach’s α = .92).
- Symptoms of Social Phobia: Symptoms of SP were measured with the 3-item Social Phobia Inventory (Mini-SPIN; [35]). Items are rated on a 5-point scale, and scale scores range from 0 to 15 (Cronbach’s α = .86).
- Symptoms of Generalized Anxiety Disorder: We assessed symptoms of GAD with the 7-item Generalized Anxiety Disorder Scale (GAD-7; [36]), which is a one-dimensional screening instrument. Items are rated on a 4-point scale, and a total score ranges from 0 to 21 (Cronbach’s α = .86).
- Depressive Symptoms: Depressive symptoms were measured with the 10-item EPDS [37]. Items are rated on a 4-point scale, and scale scores range from 0 to 30 (Cronbach’s α = .87).

#### Instrument for the assessment of discriminant validity

- General perceived self-efficacy: We assessed general self-efficacy using the 3-item General Self-Efficacy Short Scale (German name Allgemeine Selbstwirksamkeitsskala, ASKU; [38]). Items are rated on a 5-point scale. The calculated mean scale scores range from 1 to 5 (Cronbach’s α = .92).

#### Sociodemographic and obstetric data

Participants were asked via self-report forms about socioeconomic data such as maternal age, household income and educational background based on highest school degree as well as about data on parity and prior or current pregnancy complications, such as pregnancy-related hypertension, preeclampsia, HELLP-syndrome, gestational diabetes, preterm labor, miscarriage, preterm birth, as well as maternal infections during pregnancy.

### Statistical analyses

We used descriptive statistics (*M* = mean, *SD* = standard deviation, range, percentages) to describe the study participants. To test the psychometric properties of the PRAQ-R2, first scale reliability was assessed with Cronbach’s α. Second, factorial validity was assessed with confirmatory factor analysis (CFA) based on structure equation modeling. Based on the conclusion by Huizink et al. [20] we tested a first order CFA with three correlating factors. Model fit was evaluated with χ^2^ for model fit, Root Mean Squared Error of Approximation (RMSEA), Standardized Root Mean Square Residual (SRMR) and Comparative Fit Index (CFI). Factor loadings >.30 were considered indicative of importance [39]. Additionally, explorative principal axis factoring (PAF) was conducted with oblique (promax) rotation to test, whether the items would load on the same three factors without a-priori restriction on their structure.

Finally, construct validity was evaluated using correlational analysis. In the case of not normally distributed scale scores, Spearman’s rank correlation was used. The measurements used for construct validity were assessed in 253 women. CFA was conducted with MPlus 6.11 [40] and IBM© SPSS 22 [41]. The rate of missing items was very low for PRAQ-R2 items (0.6%) and the predictor variables (≤2.4%). Thus, missings were replaced using the Expectation-Maximization imputation.

To calculate a CFA for three factors and 10 items based on a RMSEA of .05, α of .05 and a power of 80%, a sample size of 317 was required to ensure statistical power (calculated with R-package semPower; [42]). With a total sample of *N* = 360, statistical power was given.

## Results

### Sample characteristics

Overall, the cohort was well educated and had an average-to-high income and most women were in a relationship. Fifty-four percent of the women were expecting their first child. For detailed information on socioeconomic and obstetric cohort characteristics, see Table 1.

**Table 1:** Characteristics of the sample (*N* = 360)

Variable	
Maternal age in years, *M* (*SD*), range	32.75 (3.77), 20 to 44
In a relationship *n* (%)	349 (96.9)
Education, *n* (%)	
main or middle school	74 (20.6)
high school graduation	88 (24.4)
university degree	186 (51.7)
information not provided	12 (3.3)
Monthly household income, *n* (%)	
≤ 1000 €	8 (2.2)
1001–2000 €	20 (5.5)
2001–4000 €	136 (37.8)
≥ 4001 €	173 (48.1)
information not provided	23 (6.4)
Ethnic background, *n* (%)	
Central European	344 (95.6)
Arabian	5 (1.4)
Eurasian	3 (0.8)
Asian	3 (0.8)
information not provided	5 (1.4)
Gestational age in weeks, *M* (*SD*), range	38.59 (1.81), 31 to 42
Expecting first child, *n* (%)	195 (54.2)
Complications, *n* (%)^:^	
Women reporting complications in current pregnancy^a^	66 (18.3)
Gestational diabetes	18 (5.0)
Maternal infections during pregnancy, not pregnancy-related	10 (2.8)
Pregnancy-related hypertension	9 (2.5)
False labor	7 (1.9)
Preeclampsia	6 (1.6)
HELLP-Syndrome	3 (0.8)
Others	16 (4.4)
Women reporting complications in previous pregnancy^b^	19 (5.3)
previous miscarriage	73 (20.3)

^a^multiple answers possible ^b^ history of preterm labor or preterm birth and previous complications as listed in ^a^

### PRAQ-R2 item characteristics

Item means were low to medium (range: *M* = 1.70 to 2.83), with overall low standard deviations (range: *SD* = .87 to 1.08), and items were partly left-skewed (range: 0.10 to 1.38); values for kurtosis varied (range: 0.01 to 1.55). According to Kline [43], the distribution of item scores was appropriate for the subsequent analysis. Cronbach’s *α* for the PRAQ-R2 total score was high with .85. All item-intercorrelations were statistically significant. Cronbach’s α for the subscales were satisfactory to excellent (FoGB: α = .77, WaHC: α = .90 and CoA: α = .89). PRAQ-R2 item characteristics are listed in Table 2.

**Table 2:** PRAQ-R2 item characteristics and values of item reliability (*N* = 360)

	*M*	*SD*	Total-item correlation	Cronbach’s *α*, if item was deleted
Fear of giving birth	7.26	2.53		
Item 1 - worry about pain	2.83	.99	.53	.84
Item 2 - anxious about delivery	2.53	1.08	.54	.84
Item 3 - worry about losing control	1.71	.98	.53	.84
Fear of bearing a physically/mentally handicapped child	8.41	3.50		
Item 4 – child mentally handicapped	2.29	1.06	.61	.83
Item 5 – perinatal death of child	2.01	1.04	.56	.84
Item 6 – physical defect of child	2.20	.96	.60	.84
Item 7 – child in poor health	1.70	.87	.60	.84
Concerns about own appearance	6.30	2.90		
Item 8 – not regaining figure	2.06	1.02	.53	.84
Item 9 – unattractive appearance	2.01	1.02	.57	.84
Item 10 – weight gain	2.08	1.08	.51	.84
Total score	21.97	6.74		

The score for each item ranges from 1 to 5

### Factorial validity

**Fig. 1:**
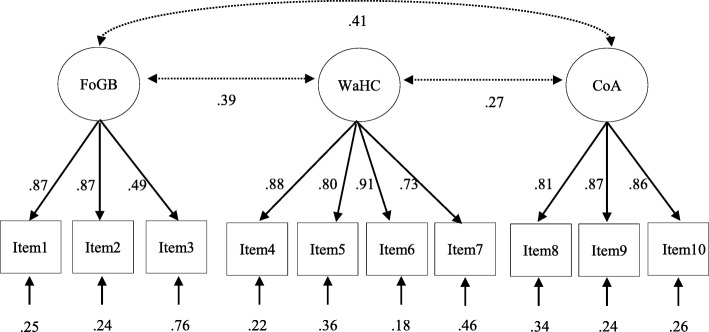
Factor structure of PRAQ-R2

Confirmatory factor analysis for PRAQ-R2 showed an acceptable-to-good model fit in the sample, χ^2^ (32)=98.539, *p* < .01, CFI = .97, TLI = .96, RMSEA = 0.08 (90% CI .06, .09), SRMR = .06. Standardized factor loadings are presented in Fig. 1. Low- to medium-sized correlations on the subscale level confirmed the three-factor solution of the PRAQ-R2. The factor loadings were lowest for item 3 (“I am worried about not being able to control myself during labor and fear that I will scream”, *r* = .49).

Explorative principal axis factoring with an unrestricted baseline estimation for the PRAQ-R2 revealed a satisfactory three-factor solution. Sampling adequacy was confirmed with a Kaiser-Meyer-Olkin value of .81. Correlations between items were sufficiently large for PAF. The Bartlett’s test of sphericity, χ^2^ (45)=2082.740, *p* < .001, also supported the appropriateness of PAF. Analysis with promax rotation revealed three factors with eigenvalues ≥1, which explained 68.1% of the total variance (FoGB = 10.2%, WaHC = 40.2%, CoA = 17.7%). Factor loadings were again lowest for item 3 (*r* = .31).

### Construct validity

Correlations of PRAQ-R2 total and subscales with the other instruments were all significant (Table 3). Distributions of the variables assessed for construct validity were skewed to the left for anxious and depressive symptoms (range 0.86 to 2.1) and to the right for perceived self-efficacy (− 0.92). Thus, Spearman correlations were calculated. The PRAQ-R2 total score correlated moderately with the CWS total score. On a subscale level, WaHC was strongly correlated with especially the CWS “health of the baby” subscale. Further, FoGB was strongly correlated with the CWS “socio-medical” subscale. CoA was moderately correlated with the CWS “socio-medical” subscale. In comparison, the PRAQ-R2 total score and its subscales showed the lowest correlations with the CWS “socio-economic and relations” subscale.

**Table 3:** Descriptive statistics of the variables assessed for construct validity and their correlations with the PRAQ-R2 total score and its subscales (*n* = 253)

Variable	*M*	*SD*	PRAQ-R2			
			Total	FoGB	WaHC	CoA
CWS-total score	0.87	0.53	.55**	.44***	.50***	.27***
socio-medical	1.12	0.78	.60***	.59***	.45***	.30***
socio-economic	0.70	0.65	.18**	.12*	.13*	.17**
health of the baby	1.14	0.93	.60***	.33***	.77***	.18**
health of mother/ others	1.08	0.97	.27***	.16**	.27***	.11*
STAI-S	34.15	0.77	.40***	.30***	.38***	.18***
STAI-T	34.52	8.81	.40***	.28***	.34***	.22***
Mini-SPIN	2.85	2.74	.29***	.25***	.16**	.24***
GAD-7	3.90	3.44	.39***	.28***	.33***	.21**
EPDS	5.31	4.87	.44***	.32***	.35***	.26***
ASKU	4.19	0.63	−.30***	−.24***	−.28***	−.15**

*p* < .05; **p < .01; ***p < .001;

* Instruments: *CWS* Cambridge Worry Scale, *STAI-S/ STAI-T* State-Trait Anxiety Inventory, *Mini-SPIN* Short version of the Social Phobia Inventory, *GAD-7* 7-item Generalized Anxiety Disorder Scale, *EPDS* Edinburgh Postnatal Depression Scale, *ASKU* General Self-Efficacy Short Scale

Furthermore, the PRAQ-R2 total score showed significant positive associations with state anxiety, trait anxiety and symptoms of GAD and the strongest associations with depressive symptoms.

Of the subscales, WaHC showed the highest correlations with these instruments, which were moderate-sized. The lowest correlations with these instruments were reported for CoA.

A different pattern was found for symptoms of SP. The PRAQ-R2 total score and WaHC showed the lowest correlations with symptoms of SP compared to FoGB and CoA.

As expected, the PRAQ-R2 total score and subscales correlated negatively with general self-efficacy. On the subscale level, the strongest medium-sized association was again reported for WaHC.

### Additional analysis of a 9-item version of the PRAQ-R2

Since item 3 showed the overall lowest factor loading in confirmatory and exploratory analysis, an alternative 9-item version of the PRAQ-R2 without this item was tested. BIC and AIC indicate a slightly better model fit (for details see Appendix). The 9-item version explained 72% of the variance in the sample compared to the 68% of the original 10-item PRAQ-R2.

## Discussion

The aim of this study was to assess the psychometric properties of the German translation of the adapted PRAQ-R2 in a sample of in total 360 parous and nulliparous women in the last trimester of pregnancy. Reliability for the PRAQ-R2 total score and its subscales was confirmed for women in the third pregnancy trimester. The three-factor structure assessing birth- as well as child- related worries and concerns regarding mother’s appearance and bodily changes during pregnancy was replicated. The subscales FoGB, WaHC and CoA together explained 68% of the total variance.

Both confirmatory and exploratory factor analysis showed satisfactory model fit, which is in line with the results reported in the validation studies of both PRAQ-R [20] and PRAQ-R2 [21]. Factor loadings of the individual items on their specific factor were high and very similar to Huizink et al. [20]. Thus, PRAQ-R2 showed high factorial validity in our sample. Further, item means and standard deviation were overall comparable for the nulliparous and parous women assessed at week 34 in the original validation sample [21]. Regarding factorial validity, our results are further comparable to the psychometric properties of the PRAQ-R2 in French [22] and Turkish [23] samples. However, regarding item means, the French and Turkish women assessed in these studies scored higher on item level [23] and in the subscales and total score [22], respectively. Further research investigating these aspects more carefully might clarify, whether these differences are systematic and caused by specific underlying factors.

As in the original validation studies by Huizink et al. [20, 21], item 3 (“I am worried about not being able to control myself during labor and fear that I will scream”) showed the lowest mean scores, the lowest factor loadings and highest error variances. For the French and Turkish sample of multiparae women, this item also showed the lowest factor loadings [22, 23]. Model fit indices indicate a slightly better fit for the 9-item version, with an overall higher percentage of explained variance. While the percentage of explained variance decreased for FoGB, the percentage of explained variance increased for WaHC and CoA. Nevertheless, the reported associations with symptoms of SP indicate that item 3 might be relevant in women experiencing specific forms of anxieties. Further analyses of the PRAQ-R2 in more diverse or high-risk samples could highlight the background of our results. Therefore, we decided to keep this item, despite the slight improvements in model fit of a 9-item version. Convergent validity was confirmed by positive associations between PRAQ-R2 and CWS, which were highest between FoGB and the CWS “socio-medical” subscale as well as between WaHC and the CWS subscale “health of baby”. These results indicate that both instruments are suitable to assess child- and birth-related concerns besides other relevant topics. Nevertheless, the size of the correlation coefficients indicates that the PRAQ-R2 is not redundant to the CWS but a valuable addition for a brief and focused assessment of PrA. Moreover, the PRAQ-R2 is able to assess maternal concerns about bodily changes in pregnancy, which is a unique feature of this instrument in comparison to other measurements of PrA [14].

Further, positive but only low- to medium-sized associations were reported with depression and state and trait anxiety as well as with symptoms of GAD and SP. These results are in line with previous studies [24–26] and support the assumption of pregnancy-related anxiety as an independent construct. On a subscale level, WaHC showed the strongest associations with symptoms of GAD, which is consistent with previous findings [3]. Symptoms of SP were less associated with the PRAQ-R2 total score and its subscales compared to other forms of anxiety. Only few studies have focused on the associations of PrA and SP thus far. Martini et al. [28] showed that women with clinically diagnosed SP more often indicated their child-related anxiety after birth as excessive. Unfortunately, due to different study designs and measurements, the results are not sufficiently comparable. Thus, the association between prenatal SP and different forms of PrA, including bodily and child-related concerns before birth, needs to be the focus of further studies. Discriminant validity was confirmed by negative associations between perceived self-efficacy and PRAQ-R2 total and subscales. As expected, higher perceived general self-efficacy was associated with lower pregnancy-related anxiety, which is consistent with findings of Lowe [31] or Salomonsson, Gullberg [44]. Since previous studies often focused on fear of childbirth and birth-related self-efficacy, our results expand these results to other aspects of PrA and show that general self-efficacy is also negatively associated with child-related worries and concerns about own appearance. Among the strengths of our study are the sample size, the involvement of parous and nulliparous women as well as the population-based design. Our population-based sample is comparable to the general population regarding the percentages of prior miscarriages [45] as well as the prevalence of current pregnancy complications [46, 47].

Besides the strengths of our study, there are also some limitations to consider. First, the sample was rather homogenous regarding relationship status and socioeconomic background, which might limit the generalizability of our results. Second, since participation was voluntary and without financial compensation, we cannot rule out a selection bias in our sample. Third, the exclusion criteria applied in our study may have led to the exclusion of women who potentially are at risk of having higher levels of PrA. Therefore, the reported values of PrA might be an underestimation of PrA in the overall population of German pregnant women.

Thus, our findings should be replicated in a more diverse sample of similar sample size including women with high-risk pregnancies, in particular regarding the relevance of item 3. Further, the psychometric properties of the German version should be investigated at earlier stages of pregnancy to confirm measurement invariance reported in the original questionnaire [21]. Moreover, it would be interesting to investigate the predictive validity of the PRAQ-R2 in cross-cultural longitudinal studies, for example regarding its relation to worries about the child’s development or health postpartum.

## Conclusion

The German Pregnancy-Related Anxiety Questionnaire, revised for parous and nulliparous women (PRAQ-R2), enables a feasible and reliable assessment of prenatal anxiety related to this particular pregnancy, the health of the unborn as well as labor and childbirth. Our study confirms the high factorial and construct validity of the three-factor solution of the PRAQ-R2 in a sample of German-speaking women in the last trimester of their pregnancy. The German PRAQ-R2 can serve as a suitable and valid measurement of pregnancy anxiety, for clinical and scientific purposes.

## Data Availability

The datasets analyzed in this study are not publicly available according to the ethical committee’s decision. For more information on the data please contact the corresponding author.

## Appendix

**Table 4:** Comparing results of CFA and PAF in original 10-item version of the PRAQ-R2 and an alternative 9-item version after excluding item 3

	10-item version	9-item version
CFA		
χ^2^ (*df*)	98.54 (32)	55.66 (24)
*p* (χ^2^)	.00	.00
CFI/TLI	.97/.96	.98/.98
RMSEA (90% CI)	.08 (.06, .09)	.06 (.04, .08)
*p* (RMSEA ≤0.05)	.01	.19
SRMR	.06	.03
AIC/BIC	8446.28/8317.98	7391.57/7508.15
PAF		
KMO	.81	.79
Bartlett’s test of sphericity χ^2^ (*df*)	2082.74 (45)	1958.71 (36)
*p* (χ^2^)	.00	.00
Explained variance (%)		
FoGB	10.16	11.16
WaHC	40.24	41.60
CoA	17.69	19.60
PRAQ-R2 total score	68.09	72.34

*KMO* Kaiser-Meyer Olkin criterion of sampling adequacy, *AIC* Akaike’s Information Criterion, *BIC* Schwarz’s Bayesian Criterion

## Abbreviations

ART: Assisted reproductive technology
ASKU: General Self-Efficacy Short Scale
CFA: Confirmatory Factor Analysis
CFI: Comparative Fit Index
CoA: Concerns about own appearance
CWS: Cambridge Worry Scale
EPDS: Edinburgh Postnatal Depression Scale
FoGB: Fear of giving birth
GAD: Generalized anxiety disorder
GAD-7: 7 item-Generalized Anxiety Disorder Scale
MINI-SPIN: Short version of the Social Phobia Inventory
PAF: Principal axis factoring
PrA: Pregnancy-related anxiety
PRAQ-R2: Pregnancy-Related Anxiety Questionnaire-Revised
RMSEA: Root Mean Squared Error of Approximation
SP: Social phobia
SRMR: Standardized Root Mean Square
STAI: State-Trait-Anxiety Inventory
WaHC: Worries of bearing a physically or mentally handicapped child
